# Draft Whole-Genome Sequence of *Xenorhabdus* sp. Strain GDc328, Isolated from the Indigenous South African Nematode Host *Steinernema khoisanae*

**DOI:** 10.1128/genomeA.01239-15

**Published:** 2015-10-22

**Authors:** Lee-Anne O. Soobramoney, Jonathan Featherston, Vincent M. Gray

**Affiliations:** a School of Molecular and Cell Biology, University of the Witwatersrand, Johannesburg, South Africa; b Agricultural Research Council Biotechnology Platform, Pretoria, South Africa

## Abstract

Here, we describe the draft genome sequence of *Xenorhabdus* sp. GDc328, an endosymbiont of the native South African entomopathogenic nematode host, *Steinernema khoisanae*. The total genome size of the bacteria is 4.09 Mb. The genome comprises a total of 3,608 genes with a molecular G+C content of 44.64%.

## GENOME ANNOUNCEMENT

*Xenorhabdus* species are Gram-negative enteric gammaproteobacteria. These bacteria display a rod-shaped morphology and are motile microorganisms (1). *Xenorhabdus* species are facultative anaerobes with respiratory and fermentative forms of metabolism (2). One remarkable attribute is their mutualistic symbiotic association with entomopathogenic nematodes (EPNs) in the genus, *Steinernema* (1). The bacteria are hosted in specialized intestinal vesicles within the infective juveniles (IJs) of different *Steinernema* species. Following the process of entry into the insect host, the IJs release their symbiotic bacteria into the nutrient-rich hemolymph of the insect (3). Subsequently, the bacteria proliferate and ensure rapid insect host mortality within 48 h. *Xenorhabdus* species have demonstrated the ability to evade the insect through the inhibition of host immune responses. A hierarchy of virulence factor regulation has been observed in different *Xenorhabdus* species which implied the stringent control of gene expression (3). Here we describe the draft genome sequence and annotation of *Xenorhabdus* sp. GDc328 isolated from the native South African species *Steinernema khoisanae* (GenBank accession no. KM275351).

*Xenorhabdus* sp. strain GDc328 was isolated from the host, *Steinernema khoisanae* according to the methods described by Akhurst (4). The genomic DNA of the bacteria was isolated from freshly streaked 24-h bacterial colonies using the ZR Genomic DNA tissue miniprep kit (Zymo Research, catalogue #D3050). The genomic bacterial DNA was purified using the ZR bacterial DNA Clean & Concentrator-5 kit (catalogue #D4013). The genomic DNA of the bacteria was quantified using NanoDrop-1000 spectrophotometer (Thermo-scientific). Paired-end genomic DNA libraries were generated and indexed using the Nextera DNA sample preparation kit (Illumina) and the Nextera index kit (Illumina), respectively. Paired-end sequencing (2 × 300 bp) was performed on the MiSeq Illumina Platform using the MiSeq reagent kit v3 at the Agricultural Research Council (ARC) Biotechnology Platform. Quality and adapter trimming of the reads was performed using the Trimmomatic0.32 algorithm. The reads were trimmed using the ILLUMINACLIP and MINLEN trim options.

A total of 1,371,386 sequence reads (Paired-end) with coverage of 99× was generated from this algorithm. A *de novo* genome assembly was performed using the prokaryotic SPAdes3.5 genome assembler. The assembly comprised a total of 300 contiguous fragments (largest, 204,218 bp) with a *N*_50_ value of 54,354 bp. The total genome size was 4.09 Mb with a molecular G+C content of 44.64%. These findings were coherent with similar *Xenorhabdus* whole-genome shotgun projects (5). The genome annotation was achieved using the NCBI Prokaryotic Genome Annotation Pipeline (PGAP). The genome of *Xenorhabdus* sp. strain GDc328 has a total of 3,959 genes. Among the identified genes, 3,608 genes were protein coding sequences (CDS) and 259 genes were pseudo genes. The genome was comprised of 17 rRNA genes (5S, 16S, 25S) and 72 tRNA genes. The FlhA and FlhB genes together with the transcriptional regulator Lys-R has been identified in *Xenorhabdus* sp. GDc328 and has been previously implicated in the enhanced virulence of many *Xenorhabdus* species (3, 6).

### Nucleotide sequence accession numbers.

This whole-genome shotgun project has been deposited at DDBJ/EMBL/GenBank under the accession no. LGYQ00000000. The version described in this paper is version LGYQ01000000.
